# Supplementary material for: Smooth Interpolating Curves with Local Control and Monotone Alternating Curvature
Source: Comput Graph Forum. 2022 Oct 6;41(5):25–38. doi: 10.1111/cgf.14600 (PMC9827861; doi:10.1111/cgf.14600)
Supplement: Supplementary file 1 — Supplement Material [file CGF-41-25-s001.zip › Local-Smooth-Interpolating-MonoCurvature/extern/clothoids/submodules/Utils/docs/zstr.html]

Zstr — Utils v1.2

### Navigation

- index
- toc
- next
- previous
- Utils »
- Zstr

# Zstr¶

Note

A C++ ZLib wrapper by Matei David,
published on GITHUB
here.

STL iostream implementation using the library zlib.
This means that you can easily manipulate zipped
streams like any other STL ostream/istream.

To give you an idea, consider following snippet
that create a gzipped files:

```
std::ofstream file("hello_world.txt.gz");
zstr::ostream gzfile(file);
gzfile << "Hello world\n";
gzfile.flush(); // must be flushed before close stream
file.close();
```

Note

From original documentation:

As you can see adding zipped buffers into your existing applications
is quite straightforward.

### Quick search

### Table of Contents

- rang - Colors for your Terminal
- Zstr
- C++ API

«
hide menu

menu
sidebar
»

### Navigation

- index
- toc
- next
- previous
- Utils »
- Zstr

© Copyright 2021, Enrico Bertolazzi.
Created using Sphinx 4.2.0.
